# Governance of Intersectoral Collaborations for Population Health and to Reduce Health Inequalities in High-Income Countries: A Complexity-Informed Systematic Review

**DOI:** 10.34172/ijhpm.2022.6550

**Published:** 2022-02-23

**Authors:** Elizabeth Such, Katherine Smith, Helen Buckley Woods, Petra Meier

**Affiliations:** ^1^School of Health and Related Research, University of Sheffield, Sheffield, UK.; ^2^University of Strathclyde, Glasgow, UK.; ^3^Research on Research Institute, University of Sheffield, Sheffield, UK.; ^4^MRC/CSA Social and Public Health Sciences Unit, Institute of Health and Wellbeing, University of Glasgow, Glasgow, UK.

**Keywords:** Intersectoral Collaboration, Health in All Policies, Healthy Public Policy, Health Inequalities, Health Equity, Governance

## Abstract

**Background:** A ‘Health in All Policies’ (HiAP) approach has been widely advocated as a way to involve multiple government sectors in addressing health inequalities, but implementation attempts have not always produced the expected results. Explaining how HiAP-style collaborations have been governed may offer insights into how to improve population health and reduce health inequalities.

**Methods:** Theoretically focused systematic review. Synthesis of evidence from evaluative studies into a causal logic model.

**Results:** Thirty-one publications based on 40 case studies from nine high-income countries were included. Intersectoral collaborations for population health and equity were multi-component and multi-dimensional with collaborative activity spanning policy, strategy, service design and service delivery. Governance of intersectoral collaboration included structural and relational components. Both internal and external legitimacy and credibility delivered collaborative power, which in turn enabled intersectoral collaboration. Internal legitimacy was driven by multiple structural elements and processes. Many of these were instrumental in developing (often-fragile) relational trust. Internal credibility was supported by multi-level collaborations that were adequately resourced and shared power. External legitimacy and credibility was created through meaningful community engagement, leadership that championed collaborations and the identification of ‘win-win’ strategies. External factors such as economic shocks and short political cycles reduced collaborative power.

**Conclusion:** This novel review, using systems thinking and causal loop representations, offers insights into how collaborations can generate internal and external legitimacy and credibility. This offers promise for future collaborative activity for population health and equity; it presents a clearer picture of what structural and relational components and dynamics collaborative partners can focus on when planning and implementing HiAP initiatives. The limits of the literature base, however, does not make it possible to identify if or how this might deliver improved population health or health equity.

## Background

 A sizeable body of evidence has sought to understand, explain and contribute to reducing health inequalities.^1^ Yet many countries chart persistent health gaps between their most and least marginalized groups.^2,3^ Although some more positive accounts have recently emerged in Western Europe,^4,5^ there has nonetheless been much criticism of, and reflection about, the apparent lack of success in reducing social gradients in health outcomes.^3^ While some of these critiques emphasize the limitations of the evidence base, many point to the failure of political and policy actors to respond to available evidence.^6-8^ Available assessments suggests that at least part of the problem has been the complex, cross-cutting nature of health inequalities:

 “Health inequalities […] have more of the character of a ‘wicked problem’ and more in common with complex systems [than health services] […] Wicked problems cut across traditional service and organisational boundaries and demand a whole system perspective.”^9^

 It is in the context of an awareness about the broad, cross-cutting nature of the determinants of health (and health inequalities) that Finnish policy-makers experimented with, and subsequently promoted, an approach they termed ‘Health in All Policies’ (HiAP). This involves working to ensure that all policy areas start to recognise their role in health, to value health and wellbeing, and contribute to enhancing population health.^10^ Yet, the effectiveness of intersectoral collaborations for reducing health inequalities remains disputed and historical experience of attempts to join up functionally divided government teams is discouraging.^11^ Earlier reviews have identified both an absence of robust evidence of effects or, at best, weak evidence of positive effects of intersectoral collaboration on health and equity outcomes.^12-15^ Multiple methodological, conceptual, theoretical, political and policy challenges have been noted to inhibit efforts to both achieve and evidence the effects of HiAP.^15^

 This troubling backdrop does not appear to have undermined enthusiasm for ‘whole government’ or collaborative approaches to achieving healthier public policy, with evidence of ongoing commitments across global, national and local policy levels.^16-19^ While challenges persist on tracking HiAP action to health and health equity outcomes, there is a large but disparate evidence base describing, assessing, theorising and evaluating a multitude of HiAP-approach initiatives. This body of research suggests a complex intersection of critical contextual factors can promote or impede the initiation and implementation of healthy public policies and intersectoral working.^20-23^ Implementation factors include the political prioritisation (or not) of HiAP,^24^ formal implementation processes, the availability of resources and capacity building activities.^20^ In addition, interprofessional trust and meaningful stakeholder and community engagement have been identified as important to the implementation of HiAP.^25-27^

 The processes of partnership working for health have also been scrutinised. Corbin and colleagues’ scoping review of what makes intersectoral partnerships for health promotion work, identified nine core elements of partnership processes that could inform best practice: (1) develop a shared mission aligned to the partners’ individual or institutional goals; (2) include a broad range of participation from diverse partners and a balance of human and financial resources; (3) incorporate leadership that inspires trust, confidence and inclusiveness; (4) monitor how communication is perceived by partners and adjust accordingly; (5) balance formal and informal roles/structures depending upon mission; (6) build trust between partners from the beginning and for the duration of the partnership; (7) ensure balance between maintenance (activities that keep partnerships functioning in practical ways) and production (activities that deliver on objectives); (8) consider the impact of political, economic, cultural, social and organizational contexts; and, (9) evaluate partnerships for continuous improvement.^28^ To enable positive collaborative processes and to give them a specific form and function, tools, most notably health impact assessments (HIAs) and health equity impact assessments, have been identified as assisting the implementation of HiAP. HIA is a practical approach to judge the health effects of policy. They are used as a way of informing decision makers and stakeholders about the health harms and benefits of specific policy interventions. They are an aid to decision making and central to some HiAP-style initiatives. Some of the focused literature seeking to understand how HIAs function in practice suggests that the way in which such tools are used and, therefore, their role in supporting meaningful intersectoral collaboration to achieve health outcomes, can also be limited by wider politico-administrative factors.^29^

 In sum, the growing research field of intersectoral collaboration for health in general and of HiAP in particular have resulted in several efforts to review and synthesise the burgeoning literature, with a view to identifying the lessons for policy.^30-32^ One of the most recent reviews reports that the available literature often finds a disappointing gap between HiAP expectations and outcomes, noting that much of this literature constitutes ‘policy analysis (identifying policy problems and solutions) rather than policy theory (explaining policy-making dynamics).’^30^ Our scoping research reached a similar conclusion and informed a decision to drop one of our original questions exploring what the existing literature suggests about the consequences of intersectoral collaboration for outcomes in policy, practice and health (in sum, the existing literature says very little about this important question).

 Building on these observations, our review specifically focuses on identifying: (*i*) the components and dynamic interactions involved in intersectoral collaboration to improve population health outcomes in policy settings; (*ii*) plausible theories of change connecting intersectoral collaboration to population health outcomes (which might inform future research focusing on outcomes); and (*iii*) insights into sustaining successful intersectoral collaborations for population health over time within policy settings. To aid our analysis, we draw on two key concepts: policy governance and systems thinking. The concept of governance has been variously defined^33^ but we use it to focus our attention on analysing the bureaucratic processes, rules (formal and informal), structures and relationships that existing evidence suggests are important for understanding intersectoral collaboration for population health. Then, informed by systems thinking, we focus on trying to identify any non-linear, unpredictable dynamics that have been identified as important for the creation and operation of intersectoral (often multi-level) policy systems for population health. To aid this part of our analysis, we draw on our findings to develop a causal loop diagram of intersectoral collaboration for population health.

## Methods

 We draw on literature on how intersectoral collaboration for health/equity is constructed and operates in practice. Although much of this literature specifically uses the HiAP concept, we wanted to ensure that we did not exclude literature that did not employ this term but nonetheless involved intersectoral collaboration with the intention of achieving population health outcomes. It incorporates a theoretical focus by working to identify and synthesise theories of change that link intersectoral collaboration to population health outcomes. As outlined above, it is also informed by two key concepts: policy governance and systems.^34^ As such, the review acknowledges that collaborative working to address health inequalities involves complex interactions, with a view to addressing a complex problem. While the review methodology included mechanisms for identifying health outcomes, early scoping and several existing reviews noted that outcomes of intersectoral collaboration for health and equity were hard to discern and inconclusive.^12-15^ In response, this review employed a methodology that enabled examination of ‘what happens’^35^ in collaborations; an approach which involved identifying the component parts of intersectoral collaboration for population health and then working to establish the causal connections between these attributes (components) and processes (dynamics). We synthesise this aspect of our analysis visually, in a causal loop diagram. Although this work is not yet sufficient for understanding the consequences of different approaches to intersectoral collaboration for population health, by surfacing some of the pathways through which collaborations function in their distinct settings, and identifying theories of change within existing literature, we believe our review provides an important first step in shifting the focus of research from processes to outcomes. The review protocol is registered on PROSPERO^36^ (reference number CRD42019138779).

###  Search Strategy and Selection Criteria

 The search was conducted across eight databases: Medline, Embase, Social Policy and Practice, Web of Science, IBSS, ASSIA + Sociology Abstracts and the Sociology Database (PROQUEST Sociology Collection), Scopus and PROQUEST dissertations and theses.

 Specific and controlled vocabulary (eg, MESH terms) were used in the search. Examples of search terms for specific databases are included in Supplementary file 1. Searches were limited to publications after 2000. Two searches were conducted; the first in October 2019, followed by a final search using the same criteria in June 2021.

 All primary studies that had an evaluative component were included as case studies. Further selection criteria were:

###  Inclusion Criteria

- National, regional, local intentional collaborations between two or more government policy areas (here, referred to as ‘sectors’), with or without other sectoral (eg, third sector) collaborations;
- Explicit population health or health equity target outcomes intended to prevent inequities in health before they become clinically identifiable (ie, not merely increasing access to healthcare);
- Empirical evidence on the component parts and dynamics of intersectoral collaboration.

###  Exclusion Criteria

- Collaborations *within* one area (sector) of government eg, collaborations between different professionals within the same sector only, such as healthcare practitioners and public health professionals;
- Collaborations between a single policy area (sector) and research/academic institutions;
- Collaborations between policy and community organisations only (eg, Community Participatory Action Research);
- Collaborations to improve access to healthcare only;
- Commercial/private sector and public sector collaborations (eg, UK’s Public-Private Partnerships) and legal/medical partnerships;
- Collaborations in humanitarian emergencies;
- Papers that identified the components of collaboration but did not provide insight into how those component parts interrelated;
- Studies based within lower- or middle-income countries;
- Studies of collaboration between global/international agencies (eg, United Nations agencies);
- Secondary studies including evidence syntheses and systematic reviews;
- Commentaries, book reviews, protocols, opinions, editorials;
- Papers not in English;
- Papers prior to 2000.

Application of these criteria revealed three main categories of research and evaluation: (1) empirical evaluative papers; (2) empirical descriptive papers and (3) theoretical papers. For the purposes of this analysis, only papers that empirically evaluated the success (or otherwise) of intersectoral collaboration for health equity were included. The rationale for this decision was that only evaluative papers empirically demonstrated the characteristics and dynamics of collaboration, and several also engaged with pathways along a complex causal chain in a specific context.

 Case studies were identified using Shankardass and colleagues’^20^ definition:

 “*An intersectoral initiative toward healthy public policy making, where sectors collaborate by developing policies, programmes and projects that include interventions addressing health upstream of inequities in healthcare utilization*” (2015: 467).

###  Data Extraction

 Papers were screened, downloaded and data were extracted using a template designed and tested in the software EPPI-Reviewer 4.^37^ Screening by title and abstract was undertaken by one reviewer with uncertain items referred to a second researcher. Screening at the full text phases was shared between three reviewers with uncertainties about inclusion/exclusion agreed by consensus. Two data extraction templates were devised, applied, refined and then implemented. The first was constructed in EPPI Reviewer and captured evidence of the state of the field, the components of intersectoral collaboration, collaborative dynamics and the outcomes of collaboration. Collaborative outcomes were extracted in terms of their reported success (or otherwise). The second extraction template was designed in MS Word and focussed on theories of change including inputs, mechanisms and outcomes. Extraction templates are provided in Supplementary file 2.

###  Quality Assessment

 The critical assessment of papers was informed by Dixon Woods and appraisal prompts.^38^ This technique was sufficiently flexible to allow the inclusion of a broad range of studies.^39^ No papers were excluded on the basis of quality as long as it could be discerned how the research was conducted (the methods used) and with whom (the case study). As such, the review followed the principles of others^40,41^ whereby exclusion is kept to a minimum in order to ensure fine-grained detail and potential insight was not missed. This was done in the recognition of the inherent subjectivity of the process of quality assessment in largely qualitative evidence syntheses.^42^ This also aligns with the conception of the review as seeking to identify the component parts of intersectoral collaborations for population health and to understand the dynamic relationships within this. Insight into processes and dynamics was, however, contingent on the depth of the analysis (or their richness or thickness^43^) in the papers. This represents a limitation of the review.

###  Data Analysis and Synthesis

 Data were analysed and synthesised using a critical realist-informed perspective. This helped us identify some potential causal mechanisms driving the social phenomena of collaboration.^44^ Theories of change were derived from data extracted in the MS Word template that detailed programme inputs, strategies, functions, mechanisms of action along causal chains and outcomes. Each paper was identified as a case study and guided in synthesis by the steps described by Hoon^45^ on meta-synthesis of case studies. In doing so, emphasis was placed on analysing each case study independently, then as a collection to identify strong and emergent themes across causal pathways with the objective of developing a causal loop diagram and accompanying narrative. Our process included: (*i*) identifying and collating variables in the extraction tables and mapping these onto an early stage diagram, (*ii*) interrogating the reported interconnection of factors in the extraction tables to connect variables on the emergent diagram, (*iii*) using extracted data to identify the polarity of interconnections. The process of identifying ‘higher order’ or systems level causal mechanisms across the dataset was an inductive process of interpreting repeated patterns in the evidence base of how policy level collaborations were able to effect change. An example of this method was operationalised is outlined in Supplementary file 2.

## Results

**Figure 1:**
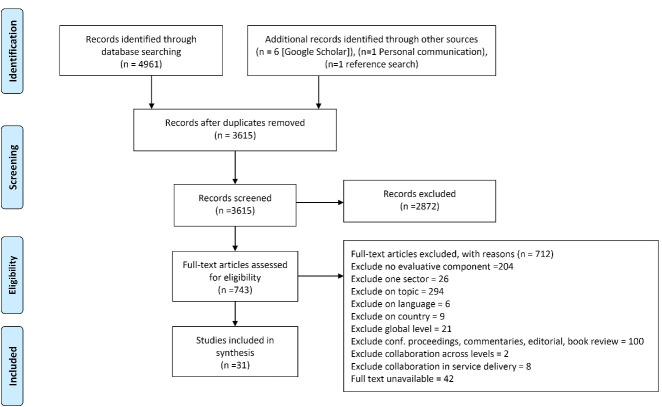
PRISMA 2009 Flow Diagram. Abbreviation: PRISMA, Preferred Reporting Items for Systematic Reviews and Meta-Analyses.

 Thirty-one publications based on 40 case studies from nine high-income countries were included in the review. The sifting process is identified in Figure 1 and summary of the papers is available in Supplementary file 3.

###  Characteristics of the Evidence Base

 Of the 31 publications included, 14 specifically focused on HiAP, using the concept explicitly to describe the approach of the policy intervention. Of those, eight were case studies of South Australia, indicating the dominance of HiAP in that context. The remaining 17 publications described intersectoral collaboration for population health and/or health equity more generally, employing a range of terms. For the remainder of this results section and where possible, we will use HiAP specifically where studies used this term.

 Reflecting the findings of other reviews, we identified only a limited evidence base assessing the effectiveness and processes of intersectoral collaborations for health and equity, which employed a narrow range of methods. Table outlines key characteristics of studies.

**Table:** Key Characteristics of the Included Case Studies

**Characteristics**		**n**
Geographic focus	The United States	6
	Australia	8
	Netherlands	3
	Denmark	3
	The United Kingdom	2
	Canada	3
	New Zealand	1
	Ireland	1
	Spain	1
	More than one country of focus	3
Methods adopted	Mixed methods	20
	Qualitative only	10
	Cross-sectional survey	1
Level of collaboration	National HiAP initiative with local implementation	1
	Regional (eg, state, county)	12
	Local (eg, community, city)	16
	Mixed levels in multiple case studies	2

Abbreviation: HiAP, Health in All Policies.

 While it is notable that over half of the case studies included the United States or Australia, many other case study locations were represented, particularly in Europe. South Australia was particularly dominant, with seven out of the eight Australian studies located there. Retrospective evaluations using only qualitative methods were common (10/31); as were mixed methods designs (20/31), including stakeholder interviews (20/20), documentary review (16/20), surveys (4/20) and other methods such as stakeholder forums,^46^ workshops,^47,48^ focus groups^49^ and analysing routine administrative data,^50-52^ progress reports^53^ and policy tracking forms.^54^

 The level of collaboration was overwhelmingly local (eg, city, community) (16/31) and regional (eg, state, region, county) (11/31) with one national collaboration initiated and then implemented at the local level.^55^ Two multiple case studies explored HiAP initiatives across policy levels.^22,56^ Collaborations for health frequently included a whole systems focus (14/31) and a wide range of policy partners were identified across the papers including planning (7/31), transport and travel (6/31), economic/urban regeneration (4/31) and housing (3/31). Less frequent but also appearing in the case studies were partnerships with the police and social services,^57^ leisure and recreation^51,58^ and immigration.^59^ Reinforcing the complexity of many of the case studies, intersectoral collaborations included not only the public sector but also voluntary and community sectors (8/31) and sometimes the private sector (2/31).

**Figure 2:**
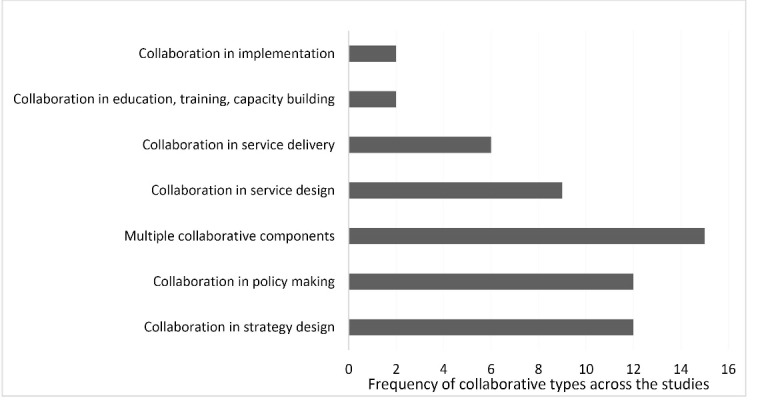
Types of Collaborative Activity Engaged in.

 A broad range of collaborative activities was identified across the studies (Figure 2), highlighting the multi-faceted nature of collaboration for health. We believe this helps underline the value of conceptualising collaboration as a system that incorporates a range of actors and relationships.

**Figure 3:**
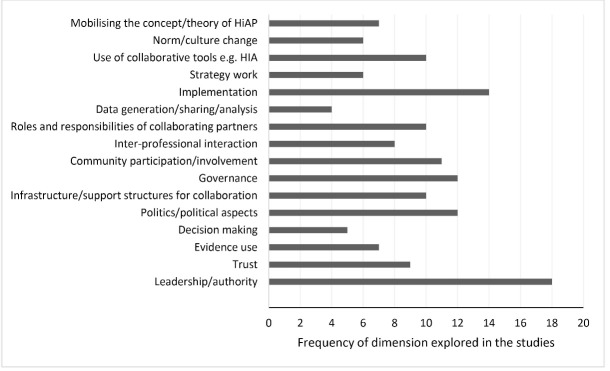
Dimensions of Intersectoral Collaboration Explored. Abbreviations: HiAP, Health in All Policies; HIA, health impact assessment.

 Multiple dimensions of collaboration, or the ways in which collaboration was manifested in the activities of collaborating partners, were explored (Figure 3). The extent to which the dimensions of collaborations intersected and related to one another were variably reported. Some papers used explicit diagrammatic frameworks (10/31) and/or textual descriptions (14/31) of how collaborations were expected to work and if and how these *a priori* understandings translated into practice.

 A range of concepts were employed to make sense of collaborative arrangements. The concept of partnerships or partnership working was most frequently applied (10/31), followed by collaborative governance^59-62^ and joined-up government.^48,49,60^ Theoretical frameworks that would help explain the process and outcomes of collaboration were few. ‘Systems thinking’ or a complex systems approach was adopted in five cases,^22,49,51,52,56^ Kingdon’s ‘multiple streams’ theory twice and social ecological theory twice.^51,54^ Fifteen papers made no reference to a theoretical framework for interpretation and analysis.

 The design of the case studies allowed only for largely qualitative outcome measures that mostly focussed on intermediate, process outcomes relating to the perceived success (or otherwise) of the collaboration itself. Examples of such outcomes included the increased references to health in strategic documents,^48,63,64^ the creation of new requirements or commitments for local governments to report on HiAP,^64,65^ the appointment of additional, sometimes boundary spanning, employees,^54,63,66^ the development of more tailored intersectoral projects^58^ and the creation of additional on-line supportive networks^66^ or decision support tools.^55^

 Programme-level outcomes reporting was partial or ambiguous in most cases. Only four evaluations identified that collaborative outcomes had been largely achieved^47,51,55,61^ but these judgements were based on divergent ‘success’ criteria. In 23 cases, activities that indicated partial success in collaboration were highlighted. One study noted that collaboration had been largely unsuccessful^67^ and in the case of three papers, it was unclear or unknown if the collaboration itself had been successful.^46,62,68^ Given these mixed findings, it is unsurprising that 21/25 papers did not identify if the collaborations had impacted population health or health equity, in line with the findings of other recent reviews.^15,30^ Two studies concluded that health equity outcomes had largely not been achieved.^69,70^ Five papers reported partial outcomes, with logic provided about how measured outcomes were consistent with steps along a causal pathway to reduced inequities.^51-53,64,71^

###  Understanding Causal Pathways

**Figure 4:**
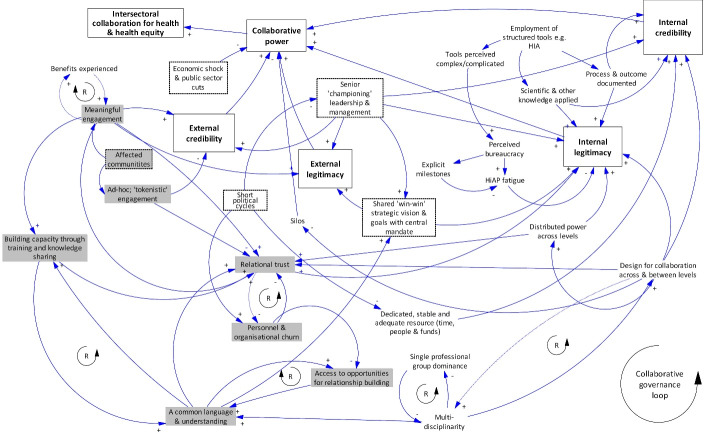
Causal Loop Diagram of Collaborative Governance of Health in All Policies. Abbreviations: HiAP, Health in All Policies; HIA, health impact assessment.
**Key:** Shaded Boxes – relational governance factors; Unshaded Boxes – structural governance factors; Dashed Outline – includes a political component; Outlined Boxes – main mechanisms/components of intersectoral collaboration.

 We sought to elucidate the causal pathways through which health and other sectors collaborate to address population health and equity via the body of included case studies. The diversity of dimensions explored (Figure 3) and divergence in the types of collaborative activity engaged in (Figure 2) provides a rich base from which to draw. We used these aspects of our findings to develop an emergent model of the components and dynamics of intersectoral collaboration – a collaborative governance loop – which is represented in Figure 4 (consult Supplementary file 2 for an explanation of the process of generating the causal loop diagram). The complexity of the collaborative picture and the limitations of the evidence base (as noted above) mean that Figure 4 is likely to be an incomplete depiction of causal pathways but we believe it is useful in visually representing the causal pathways evident in the existing evidence base.

 The collaborative governance loop represents the components of intersectoral collaboration and their interconnections. It shows the variables contributing to collaboration, linked by arrows. Links connected with a + (plus polarity symbol) show that variables move in the same direction, for example, the presence of a common language and understanding leads to more relational trust. Links with a – (minus polarity symbol) indicate the opposite, for example, the occurrence of personnel and organisational churn leads to less relational trust. Loops indicated with an R are reinforcing loops. An example in this causal loop diagram is the relationship between capacity building and the creation of a common language: more of one, means more of the other and so on.

 A variety of factors act recursively; feeding back to amplify or dampen aspects of collaboration along the causal chain. Components of the pathway to collaboration are structural (boxes without a border) or relational (shaded in grey). Structural governance components are those elements of the system intentionally put in place to govern collaborative effort. These include resources, such as time and money, as well as structural inputs, such as training, decision support tools, processes for documenting action and establishing decision-making structures. Relational governance components are the processes of the relationships between people as professionals, as representatives of organisations or institutions, as politicians or as citizens in the collaborative nexus. Some factors blend relational and structural aspects. For example, senior ‘championing’ leadership includes both the ways in which collaborations put in place a leader within a structure of governance but also the ways senior leaders relate to others across collaborations to enable the functioning of the collaborative system.

 There are also political dimensions to collaboration identified in Figure 4. In systems terms, these factors can include more distal influences (eg, changes in national political fortunes; popularity of a national or regional leader) that can be unpredictable and result in unanticipated effects.

 Internal and external credibility (the quality of being trusted or believed in) and internal and external legitimacy (the acceptance of the collaboration as an authority) are the changes anticipated to happen through lower level collaborative effort. They are the ‘higher order’ change mechanisms that are both internal to and external of the collaborations themselves. This finding is significant in that the meta-synthesis reveals that legitimacy and credibility both internal to collaborations and in the recognition they are given externally are important. These mechanisms coalesce in intersectoral collaborations for health and health equity in that they may deliver the collaborative power necessary to enable change to take effect. The notion of collaborative power is introduced to represent uncertainty about the extent to which the presence or absence of these factors (credibility and legitimacy) translate to intersectoral collaborations for health or health equity. It is not considered inevitable that credible and legitimate collaborations are capable or able to exercise the power necessary to lead to intersectoral change. Indeed, power was disputed across the case studies and fluctuated over time.

###  Structural Components and Dynamics 

 The strongest features of the evidence base on structural governance components and processes included: the role of collaborative working structures, strategies and leadership and the requirement for adequate human and financial resources and time to effect long-term change. Eighteen papers reported that formal structures were put in place to encourage collaboration between participating partners. In addition, formal leadership structures were embedded across the interventions, although these varied in form, scale and contributing partners. In South Australia, for example, several studies noted that a dedicated HiAP Unit had been created alongside the implementation of a joint governance structure that created an ‘authorising environment’ for intersectoral action.^48,64,65,72^ The scale and level of advancement of the South Australia partnership was unusual across the studies; it had a central mandate, was backed by legislation and strategy and had high level political support.^64,65^ As Delaney et al noted, however, these structural supports did not ensure implementation success, with a series of factors, such as resource-constraint and personnel/leadership churn, undermining the initiative.^48^

 Other evaluations described intersectoral administrative health committees (‘Health Forums’) that included steering committee members, public health departments and a series of ‘intersectoral working groups’ concerned with different populations including children and young people, at-risk populations and ill and debilitated people.^60,69^ Carlisle described Social Inclusion Partnerships in Scotland that were organised around committee-style management board meetings.^68^ A wide range of working group, advisory group and partnership structures were evident across included studies.^22,51,52,55,60,62,67,69^ This variety, as well as the often multiple governance structures put in place for HiAP initiatives, make conclusions about optimal or most appropriate structures difficult to establish. In their assessment of two Canadian cancer prevention partnership projects (Healthy Canada by Design and Children’s Mobility, Health and Happiness), Politis et al noted that broad, inclusive formal structures were important facilitators to intersectoral collaboration but that these required time to find ‘common ground’ and identify shared objectives.^55^ Holt et al, in a study of HiAP in 10 Danish municipalities, cautioned that the two most common governance structures designed to transcend organisational boundaries – the central unit and the intersectoral committee – reproduced existing organisational problems, particularly reinforcing silos.^60^ They noted an example of a ‘matrix’ style intersectoral committee in one municipality that showed promise. This combined strategy, policy and implementation level partners in a long-term (10-year) structure that enabled strong, embedded relationships to develop over time.^60^ These findings highlight two things: first, that collaborations should be designed to span across and between levels of governance and, second, structures in themselves may be less important than the long-term relationships they enable to develop since it is these relationships that appear key for collaborative legitimacy and credibility.

 There was consistent evidence of formal leadership appointments across collaborations (16/25). These tended to include health leaders, senior system leaders (in policy and delivery) and high profile ‘champions.’^46,48,53,58,60,61,72^ Such high profile leadership positively affected the perceived internal and external credibility and legitimacy of the collaboration.^46,61,64,70^ Characteristics of perceived effective collaborative leadership were commonly described as: ‘committed,’ ‘strong’ and ‘with decision making powers.’^46,58,60,68^ In a health and housing context, where housing were the driving partners, Haigh et al identified that leaders required seniority to be perceived as effective: “They have some power (but not final decision making power), they understand the system well, often have pre-existing relationships that they can utilise and are in a position to influence the implementation of recommendations.”^46^ However, Gase et al caution that different professions within a collaboration may have contrasting expectations about policy leadership. If these expectations are not met, then the legitimacy of leadership can be eroded which, in turn, has the potential to undermine collaborative efforts.^50^ Plochg et al highlighted that narrow, self-selected leadership was damaging to collaboration,^62^ suggesting that, like collaborative structures, strong collaborative leadership should focus on how structural decisions can create, reinforce or damage the relationships collaborative partners are seeking to achieve. The value of mutlidisciplinarity, for example, was highlighted as a way to enhance collaborations through the development of a common language and trust between partners.^48,50,54,55,73^ Morteruel et al explicitly noted that the participation of people with different profiles (eg, architects, engineers, qualitative researchers) were seen as enhancing the quality of planning-related HIAs.^74^ Two studies used multiple cases to demonstrate that a common language was positively related to the adoption and implementation of ‘win-win’ strategies^22,56^; factors promoting the internal and external credibility of collaborations.

 Where a political component of leadership was considered in studies, there was evidence that political leadership and ‘drive’ were important for instigating and sustaining the prioritisation of HiAP interventions.^47,48,51,52,60,61,64,65,69,74^ Studies also pointed to some potentially problematic elements of political leadership,^49,60,69,71^ with Holt et al identifying the importance of politicians ‘leav[ing] space’ for the professional judgements of others around the implementation of interventions.^69^ This suggests a careful balance is required. As Haigh et al note, in the context of local political settings, politicians can play a useful role as advocates throughout the collaborative process^46^; in Baum and colleagues’ terms they are powerful ‘norm entrepreneurs’ who can increase the chance of the institutionalisation of HiAP.^47^ Conversely, if political priorities shifted, such as in the cases of South Australia and Spain during healthcare budgetary pressure and economic downturn, then a failure to advocate or prioritise politically can reduce collaborative power.^47,48,65,65,72,74^

 Tools that supported intersectoral collaboration were used in 17 cases, most commonly HIAs. These were reported to support internal credibility of collaborations when assessments were documented and scientific knowledge was applied.^46,50,59,66,74^ Perceived complicated and bureaucratic delays in implementing tools could, however, undermine HiAP initiatives.^75^ A mitigating strategy of embedding milestones into the process of assessment could reduce this kind of ‘HiAP fatigue.’^48,75^

###  Relational Components and Dynamics

 Relational factors refer to those elements of collaboration that provide people opportunities to form and maintain relationships across sectors. It is notable that the diagram in Figure 4 identifies multiple positive feedback loops in the configuration, suggesting the reinforcing capacity of attendance to relational aspects of governance. There appeared to be two main pathways to collaborative power in the governance of relational factors in the case examples: internal legitimacy via the route of developing relational trust and external credibility via the process of the meaningful engagement of affected communities.

 The internal legitimacy of collaborations was causally connected to partners’ access to opportunities for relationship building. There was strong evidence that creating and accessing opportunities to build relationships facilitated the development of trust between sectors and aided collaborative legitimacy and credibility.^49,51,52,55,57,61,66,74^ Practices supporting this included early sharing of work,^48,59^ having one-to-one meetings^63^ and frequent contact with collaborators.^66^ This enabled an understanding of other sectors and the creation of a common language between partners^22,59,61,67^ which further reinforced relationships. Lawless et al describe this as part of a deliberate and necessary ‘engage step’ in the collaborative process that facilitated social learning between partners.^59^ Mathias and Harris-Roxas identified that creating opportunities for health and urban design sectors to come together in the context of recognised linked agendas positively impacted partners’ feeling of ‘being in it together.’^66^ This ethos of mutuality or reciprocity was commonly identified across the studies and was positively linked to the determination of intersectoral trust, collaborative legitimacy and credibility.^49,51,52,55,57,61,66^ Trust emerged as a critical mechanism to external credibility and internal legitimacy across a broad range of contexts. In a child health network in rural Canada, McPherson et al identified that through the 13-year life of the Network, trust was developed through processes of exposure to and respect for networked collaborators, good communication between parties, interdependent working and positive peer influence.^61^ They described this as creating a ‘collective responsiveness’ that enabled on-going collaboration and reflected that trusting relationships had become deeply embedded within the Network.^61^

 Trust between people and partners could be disrupted, however, particularly during periods of personnel or organisational change. Organisational restructuring either to accommodate intersectoral collaboration^60^ or as part of broader public sector change/reform programme^48,64,71^ or as an outcome of political change was problematic for developing and maintaining trust.^52,58,76^

 Another component of collaboration that developed trust was the relational process of building capacity through training and knowledge sharing.^22,55,67,70,77^ The strongest evidence related to capacity building, training and other engagement activity with communities affected by interventions to improve health or reduce inequalities.^50-52,54,66,77^ There were nine studies from eight interventions and one multiple case study overall where community representation was explicitly part of the collaborative exercise.^22,46,51-54,58,66,77^ Meaningful community engagement developed trust but also directly impacted on the external credibility of intersectoral collaborations. To illustrate at the local policy level, Lachance et al identified that bringing diverse stakeholders together in a healthy food and community partnership in the United States acted as opportunities to learn (‘learning communities’) which built capacity and leadership skills across community and policy stakeholders, thereby developing a broader skill set to support and build further credible intersectoral projects.^51,52^ In addition, this community-driven intersectoral partnership purposefully recruited from affected communities thereby ensuring that genuine advocates for the neighborhood were represented, that capacity among residents was developed and that policy change was sustainable.^51^ Evenson et al went further, observing that in some communities that adopted active living built environment policies, residents who experienced the positive benefits of intersectoral health promotion activity became less wary of subsequent efforts, suggesting a reinforcing feedback loop.^58^ Cheadle identified that working in a small or well-deﬁned community and having a stable group of core members enabled “workable decision-making processes and finding a match between community priorities and partnership activities.”^53^

 The dangers of ‘tokenism’ were raised, however, in relation to community engagement^51,68^; a risk that could damage external credibility and, hence, collaborative power. One study noted that late inclusion of community representatives in the policy cycle and contestation about who the ‘community’ was led to conflict within the partnership, power imbalances and poorly addressed community concerns.^68^

###  Overarching Insights From the Causal Loop Mapping Approach

 The analysis revealed four important intermediate processes in collaboration that coalesced to deliver the collaborative power necessary for HiAP: internal and external legitimacy and internal and external credibility. Internal legitimacy was driven by multiple structural elements and processes, many of which were instrumental in developing relational trust. Internal credibility was supported by adequately resourced, multi-level designed collaborations, effective use of collaborative tool (eg, HIAs) and power-sharing. External legitimacy and credibility was created through meaningful community engagement, championing leadership and the adoption of win-win strategies.

 Insight from Figure 4 highlights that the evidence base on the dynamics of collaboration centres on how structural and relational components create internal legitimacy and less on the way these processes relate to the generation of external credibility and legitimacy. This is despite the revealed centrality of, for example, meaningful community involvement at the local level in the generation of an externally endorsed mandate for intersectoral collaboration for health or health equity. A variable in the model that had multiple links to many other relational and structural factors is relational trust, suggesting the exchanges that take place between people and institutions involved in the HiAP process are critical factors that inform the success or failure of collaborations. Trust was potentially fragile, however, with the diagram showing how it could be undermined in multiple ways, including during periods of political change, personnel and organisational churn and as a consequence of perceived tokenistic community engagement.

## Discussion and Conclusion

 This evidence synthesis of case studies of intersectoral collaborations for health highlights that, reflecting earlier reviews,^12-15^ we continue to lack evidence linking intersectoral collaborations to population health outcomes. Although this is disappointing, we do have a lot of evidence, and thinking, around what makes for successful intersectoral collaboration. This paper brings this knowledge together into a causal loop diagram, providing the foundations for future research to better explore the causal pathways underlying successful intersectoral collaboration and in ways that might lead to health outcomes. Our three main conclusions are:

###  1. Intersectoral collaborations for health can be seen as a complex system.

 While studies recognise the complexities of HiAP-style interventions, none of the studies applied an explicitly a complex systems lens to their analysis. The presentation of intersectoral collaboration for health and health equity in a causal loop diagram (Figure 4) has not, to our knowledge, been attempted before. We believe this contribution is useful in enabling a visual meta-synthesis of evidence across multiple contexts that articulates not only the component parts of intersectoral collaborations but also some of the dynamically interrelationships within. As we note, however, the causal loop diagram necessarily reflects the limitations of the underpinning evidence-base. Intersectoral collaborations were inconsistently reported and theorised. Some collaborations explicitly identified how they were expected to work and effect change; others reported this partially and some included only implicit theories of change. In addition, components of the system and how they interrelated were variably reported in published evaluations. Some factors and dynamics were raised infrequently, given greater or lesser importance in reporting or viewed through theoretical or conceptual lenses that were more or less critical. This makes some of the collaborative governance loop in Figure 4 uncertain. In particular, questions around who should be part of collaborations – what is the optimal disciplinary, professional and community-based mix – is underexplored. Recent explorations, for example, suggest that public health intervention evaluations can play an important role in promoting intersectoral collaborations.^78^ Initiatives such as the UK Prevention Research Partnerships and the Australian Prevention Partnership Centre advance this notion by intentionally integrating policy, practice, community and research in collaborations for healthy public policy.^79-81^ Evidence from these consortia should play an important role in the design of future intersectoral collaborations.

 The review also raises questions of if and how affected communities are involved and the extent to which (dis)benefits are experienced by them affect existing and future collaborations. This also requires further examination, especially as much of this research focused on collaborations at a local level, leaving open questions about the importance of community and stakeholder engagement for intersectoral collaboration at national or regional policy levels. A further limitation of the model presented is its level of analysis; it does not represent some of the finer, granular detail of what happens in some of the many dimensions of collaborative activity. There is some progress in this task. For example, insight into how capacity can be built through collaboration in sport and physical activity promotion in England has revealed that, at the practitioner level, mutual confidence in the abilities and intentions of partners increased trust between partners which, in turn, led to more knowledge and skill sharing.^73^ In addition, Delany-Crowe et al deconstruct the concept of ‘trust’ in joined-up government activities, highlighting how trust can bridge the gap between the known and unknown and act as a resource to stimulate action within government systems that are perceived to feature high levels of risk.^82^ In network analyses of advancing strategies for getting health and equity into policy, McGetrick et al identify the important role of policy influencer networks and the way issues in chronic disease prevention are primed and framed to effect policy-making.^83^ These dynamics of problem priming and framing, as well as coalitions of trust, are promising avenues for further exploration and mobilisation in the context of efforts to understand, and improve, intersectoral collaboration for population health.

###  2. Application of the concepts of relational and structural governance help develop a stronger understanding of the pathways to collaborative governance for health.

 The concept of governance has been applied rarely in analyses of intersectoral collaboration for population health.^84,85^ Its development – as comprising structural and relational factors – and application to the context of intersectoral collaboration has helped to develop theory on how HiAP-style interventions have worked. Notably, structural and relational factors interrelate in the causal pathway, contributing to the credibility and legitimacy of collaborations and functioning to establish collaborative power. The interdependence of factors, their reinforcing capacity and the potential of external credibility to be undermined through a failure to properly engage with affected communities all highlights the potential fragility of the realisation and sustainability of collaboration. Indeed, while relationships may be central to collaborative governance and, as McPherson et al suggest, may ‘trigger’ system change,^61^ they are inherently intangible and often unstable in the face of internal and external change. Relationships between people and organisations are also expressions of power (im)balances; a factor that requires further examination in future studies (and may have particular implications when it comes to understanding the potentially varying consequences of community and stakeholder engagement for intersectoral collaborations for health, given the varying degrees of power and resources different organisations and social groups can draw on).

 This synthesis raises further questions about governance. Many of the interventions studied aligned with the principles of ‘good governance.’^86^ These include legitimacy and voice (eg, of local citizens), direction (eg, strategic vision) and performance (eg, responsive to local need). Study findings, however, focussed little on issues of accountability and transparency, indicating possible democratic and governance deficits in intersectoral collaborations for health equity to date. Indeed, Holt et al identified that an absence of accountability mechanisms led to the deprioritsation of the goals of collaboration^60^; lack of accountability and transparency may be a barrier to healthier, cross-sectoral public policy.

###  3. Causal pathways to collaborative power have been surfaced.

 Overall, the synthesis offers a model of some of the causal pathways through which intersectoral collaborations have been governed. Until now, as Godziewski observes, there has been a dominance of examinations of intersectoral collaboration from a technocratic or structural perspective with acknowledgement, rather than analysis, of the dynamic interaction of structure and relationships.^87^ What is required now is further examination of dynamic components, particularly with reference to politics and the power dynamics of intersectoral collaboration.^88^ Recent research has applied political science and critical policy analysis techniques to identify the dilemmas, tensions, emergent properties and context driven nature of HiAP-style initiatives.^87,89,90^ Their results caution against approaching HiAP as solely a question of centrally controlled ‘public administration’ but one of ‘public policy’ that invites us to take governance dilemmas seriously.^30^ Applying systems thinking as a means of understanding policy as a dynamic, often-unpredictable system, may offer a helpful way of making sense of HiAP and offering opportunities to intervene when efforts to collaborate appear to be failing. The proposed causal loop diagram offers a starting point for future research to examine the causal pathways that underpin successful collaborative governance in the inevitably emergent, adaptive and dynamic context of policy development and implementation. Taken together, these conclusions offer a way forward for thinking about and researching intersectoral collaboration. The causal loop diagram presented here may serve as a guide to constructing a clearer, more comprehensive picture of how collaboration of for health may play out in a given context and, importantly, link to health outcomes. This would represent a much-needed shift towards research that can identify and explain if and how intersectoral collaboration for health connects to population health outcomes. This review also provides insights that may help support the development and implementation of HiAP-style initiatives, especially at the local level (eg, practitioners and policy makers may wish to attend to pathways that can support or undermine the legitimacy and credibility of collaborations, and tailor their approaches to local settings).

## Acknowledgements

 Many thanks go to Wellcome PhD students Jen-Yu Amy Chang and Jennifer Boyd. Thanks also to Suzy Paisley, ScHARR, for comments and encouragement on the final draft.

## Ethical issues

 Not applicable.

## Competing interests

 Authors declare that they have no competing interests.

## Authors’ contributions

 ES: conception and design, acquisition of data, analysis and interpretation of data, drafting of manuscript, critical revision of the manuscript for important intellectual content, obtaining funding. KS: Analysis and interpretation of data, drafting of manuscript. HBW: Acquisition of data; administrative and technical support. PM: Analysis and interpretation of data, drafting of the manuscript, critical revision of the manuscript for important intellectual content, obtaining funding.

## Disclaimer

 The views expressed are those of the authors and not necessarily those of the funders. The funders had not role... etc.

## Funding

 This work was supported by the UK Prevention Research Partnership (MR/S037578/1), which is funded by the British Heart Foundation, Cancer Research UK, Chief Scientist Office of the Scottish Government Health and Social Care Directorates, Engineering and Physical Sciences Research Council, Economic and Social Research Council, Health and Social Care Research and Development Division (Welsh Government), Medical Research Council, National Institute for Health Research (NIHR), Natural Environment Research Council, Public Health Agency (Northern Ireland), The Health Foundation and Wellcome. ES is also funded by an NIHR Knowledge Mobilisation Research Fellowship (KMRF-2017-06-ST2-003).

## Supplementary files

Supplementary file 1. Search Terms.

Supplementary file 2. Extraction Templates.

Supplementary file 3. Included Empirical Evaluative Studies and Their Characteristics.
