# Supplementary material for: Governance of Intersectoral Collaborations for Population Health and to Reduce Health Inequalities in High-Income Countries: A Complexity-Informed Systematic Review
Source: Int J Health Policy Manag. 2022 Feb 23;11(12):2780–92. doi: 10.34172/ijhpm.2022.6550 (PMC10105187; doi:10.34172/ijhpm.2022.6550)
Supplement: Supplementary file 1 — Search Terms. [file ijhpm-11-2780-s001.pdf]

Article title: Governance of Intersectoral Collaborations for Population Health and to Reduce Health Inequalities in High-Income Countries: A Complexity-Informed Systematic Review

Journal name: International Journal of Health Policy and Management (IJHPM)

Authors' information: Elizabeth Such[1]*, Katherine Smith[2], Helen Buckley Woods[3], Petra Meier[4]

[1]School of Health and Related Research, University of Sheffield, Sheffield, UK.

[2]University of Strathclyde, Glasgow, UK.

[3]Research on Research Institute, University of Sheffield, Sheffield, UK.

[4]MRC/CSA Social and Public Health Sciences Unit, Institute of Health and Wellbeing, University of Glasgow, Glasgow, UK.

(*Corresponding author: Email: e.such@sheffield.ac.uk)

Supplementary file 1. Search Terms

'Health for all'<br>'Health in all'<br>'Health and Equity in all'<br>HiAP<br>HEiAP<br>'Healthy public'<br>Health inequal*<br>Social determinant*<br>'Health impact assessment'<br>HIA<br>'Whole government approach'<br>'Whole system approach'<br>'System* change'<br>'System* transformation'<br><br>**APPROACH**	AND	Integration<br>Collaboration<br>Silos<br>Intersectoral<br>Intersectorial<br>'Joined-up'<br>Harmonisation<br>Coherence<br>Partnership*<br>Coordinat*<br><br>**WAYS OF WORKING**	AND	Polic*<br>OR<br>Government<br>OR governance<br>OR<br>'Decision mak*'<br><br>**DOMAIN**

MESH terms used where appropriate

Medline search example

Search Strategy:
----

1 (health for all or health in all or "health and equity in all" or HiAP or HEiAP or healthy public or health inequal* or social determinant* or health impact assessment or HIA or whole government approach or whole system approach or system* change or system* transformation).ti,ab. (18998)

2 *Healthcare Disparities/ or *Health Status Disparities/ (17664)

3 *Health Equity/ (652)

4 1 or 2 or 3 (34875)
5 (integration or collaboration or silo* or intersectoral* or inter-sectoral* or inter sectoral* or joined-up or joined up or harmonisation or harmonised or harmonization or harmonized or coherence or partnership* or coordinat* or co-ordinat* or co ordinat*).ti,ab. (565021)
6 *Cooperative Behavior/ (17057)
7 5 or 6 (575512)
8 (policy or policies or government* or governance or decision mak*).ti,ab. (422919)
9 exp *Policy Making/ (8760)
10 8 or 9 (427387)
11 evidence-based medicine/ or patient education.mp. or patient.mp. or Review Literature/ (4853226)
12 10 or 11 (5141490)
13 4 and 7 (2893)
14 12 and 13 (1761)
15 limit 14 to english language (1666)
16 limit 15 to yr="2000 -Current" (1562)
****
