# Supplementary material for: Governance of Intersectoral Collaborations for Population Health and to Reduce Health Inequalities in High-Income Countries: A Complexity-Informed Systematic Review
Source: Int J Health Policy Manag. 2022 Feb 23;11(12):2780–92. doi: 10.34172/ijhpm.2022.6550 (PMC10105187; doi:10.34172/ijhpm.2022.6550)
Supplement: Supplementary file 2 — Extraction Templates. [file ijhpm-11-2780-s002.pdf]

Article title: Governance of Intersectoral Collaborations for Population Health and to Reduce Health Inequalities in High-Income Countries: A Complexity-Informed Systematic Review

Journal name: International Journal of Health Policy and Management (IJHPM)

Authors' information: Elizabeth Such[1]*, Katherine Smith[2], Helen Buckley Woods[3], Petra Meier[4]

[1]School of Health and Related Research, University of Sheffield, Sheffield, UK.

[2]University of Strathclyde, Glasgow, UK.

[3]Research on Research Institute, University of Sheffield, Sheffield, UK.

[4]MRC/CSA Social and Public Health Sciences Unit, Institute of Health and Wellbeing, University of Glasgow, Glasgow, UK.

(*Corresponding author: Email: e.such@sheffield.ac.uk)

Supplementary file 2. Extraction Templates

This is an extract of the templates used for developing the causal loop diagram. Two table extracts are shown or structural governance and relational governance factors. These include inputs, mechanisms and outcomes. PLEASE NOTE this is a sub-sample of extracted papers used for methodological illustration purposes only.

Structural governance strategies, mechanisms and outcomes (structural solutions to the problem of intersectoral collaboration)

**Paper**	**Strategies - Direct inputs**								**Mechanisms at the policy level / causal chains**	**Outcomes**
	**Structural governance inputs**								**(hypothesised and/or demonstrated or implicit) (intended / unintended)**	**(demonstrated or implicit)**
	**Formal structure**	**Legislative frameworks**	**Performance management structures**	**Formal agreements**	**Funding/ resources**	**Leadership & Man't (formal)**	**Temporal/ trajectory**	**ISC tools**		
Delany et al. 2016	✓ Dedicated HiAP Unit	✓	✓ Sectoral KPIs	✓	✓ Resourced core team	✓ Senior leaders	✓ Clear timelines and	✓	✓(QI) unintended	Mixed outcomes re. success of

	✓ Joint governance structure	Multiple legislative levers		Alignment of strategic objectives			achievement milestones	Health Lens Analysis	Traditional sectoral KPIs→persistent siloed thinking→concerns about accountability structures&problem of how to justify HiAP involvement to senior managers (esp.if unsupportive)<br>✓(QI) intended. Joint governance→exposure of achievements to senior execs across org→↑likelihood of change<br>✓(QI) intended Timelines and milestones →mitigate long term HiAP 'fatigue' → facilitates continued engagement<br>*HLA finding (see Baum et al. 2019)	intersectoral collaboration
Delany et al. 2016	✓ Dedicated HiAP Unit<br>✓ Joint governance structure	✓<br>Multiple legislative levers	✓<br>Sectoral KPIs	✓<br>Alignment of strategic objectives	✓<br>Resourced core team	✓<br>Senior leaders	✓<br>Clear timelines and achievement milestones	✓<br>Health Lens Analysis	✓(QI) **unintended**<br>Traditional sectoral KPIs→persistent siloed thinking→concerns about accountability structures&problem of how to justify HiAP involvement to senior managers (esp.if unsupportive)<br>✓(QI) intended. Joint governance→exposure of achievements to senior execs across org→**↑likelihood of change**<br>✓(QI) intended Timelines and milestones →mitigate long term HiAP 'fatigue' → facilitates continued engagement<br>*HLA finding (see Baum et al. 2019)	Mixed outcomes re. success of intersectoral collaboration
Evenson et al. 2012					✓<br>Protected funding	✓<br>'Influential champion'			✓(QI) unclear if intended<br>Powerful elected or institutional partner (knowledgeable able to capitalize on larger political priorities) and/or influential public champion→guide through bureaucracy, identify resources	Partnership successful in generating resources
Gase et al. 2014	✓<br>Diversity of partners			✓<br>A 'resolution'				✓ Health Impact Assessm ent	✓ (QI) unclear if intended<br>HIA →mechanism for PH to engage in cross-sector dialogue →↑credibility of PH → pay dividends in future policy work	
Haigh et al. 2015						✓ stepwise process ;<br>✓ 'Right' people	✓Timing and timeliness (of HIA)	✓ Health Impact Assessm ent	QI. 'Scientific' stepwise HIA process→purposeful and structured→↑engagement in meetings+added legitimacy to recommendations<br>'Right people'=senior man't (Right level) →some power (influence)&understand system+have existing r'ships→can influence implementation	
Harris et al. 2014	✓<br>Health and housing partnership			✓<br>'Mandate'			✓<br>Early engagement ; Health input at important time points		QI. Early health sector engagement in Master Plan→fosters working r'ship→protects collaboration even when Plan stalls→collaborative resilience& procedural influence in non-linear process;<br>QI Absence of core organisational mandate→attention moved to pressing, urgent matters	

<u>Relational governance</u> strategies, mechanisms and outcomes (relational solutions to the problem of intersectoral collaboration)

Paper	Strategies – direct inputs						Mechanisms at the policy level / causal chains	Outcome
	Relational governance inputs						(hypothesised and/or demonstrated or implicit) (intended / unintended)	(demonstrated or implicit)
	Leadership (soft skills)	Interpersonal Trust/ Reciprocity & togetherness	Organisational culture/ ethos	Political support	Community engagement	Multi-disciplinarity		
Delany et al. 2016		✓ learning about each others' work		✓ central mandate for action		✓ Intentional multidisciplinary skill spread ✓ De-siloed language & concepts	✓(QI) intended HiAP Unit→ gave other depts access to people with different skills→facilitated collaboration<br>Early sharing of each others' work→acceptance of HiAP approach→developed facilitate relationships<br>Avoidance of sector-specific language+examining terms e,g, health/equity across sectors→facilitated development of mutual understanding and goals	Developed mutual understanding across sectors Promoted (routine?) collaboration
Evenson et al. 2012	✓ 'Vision' for active living		✓ Celebrating success		✓community inclusion		Community inclusion (valuing ideas&opinions)→ensured needs&concerns reflected in planning→partnership had credibility in community→sustainability<br>Partnership 'vision'→influenced transport dept 'culture'<br>Small successes →boost morale→ spark similar changes in other locations, & ↑demand, providing momentum.	'Better' (more tailored) projects
Gase et al. 2014					✓community inclusion	✓ different professional groups	Professions from different sectors→contrasting expectations of leadership in policy→expectations not met→questioned legitimacy of leadership →questioned value of ISC	No clear outcomes reported; some interim promising ISC outcomes (hypothesised will lead to more/better future collaborations)
Haigh et al. 2015			✓ 'proactive positioning' of partners ✓ 'shared values' and explicit goals		✓r'ships bet. prof& community stakeholders		Proactive ethos→encourages opportunity take-up→motivates collaboration	HIA 'effectiveness'

Harris et al. 2014		✓Team 'togetherness' / outsider status of 'health'	✓ 'Openness' of each partner			✓ Skills and competencies training in collaboration	QI (unclear if intended). Partners 'open' to new ideas and ways of working→ facilitated health reps into housing 'political process', & housing reps engaging and sharing information<br>QI (unintended) divide between 'health'(partners) and housing (drivers)→ familiarity during messiness →togetherness→ failure to foster good/equal r'ships at all times	Overall, collaboration of limited effectiveness (cf. unintended processes)

A worked example of how 'external legitimacy' was built from the extracted data where external legitimacy refers to the acceptance of the collaboration as an authority by organisations and people external to it.

In the causal loop diagram, there are 3 variables that relate to the development of external legitimacy: 1. The meaningful engagement of affected communities; 2. Shared 'win-win' strategic vision and goals with central mandate; 3. Senior 'championing' leadership and management.

- Relational governance inputs = community engagement (n=3 papers above)
- Relational mechanisms – Evenson 'meaningful engagement' increased legitimacy with communities
- Structural inputs = senior leadership (n=3 papers above); shared strategic vision/formal agreements (n=4 papers above)
- Structural mechanisms – Delaney: senior cross-sectoral joint governance gave exposure of achievements to senior execs across org and increased external legitimacy. Evenson and Haigh re. senior leadership – inclusion of people with power and influence across the system – increases legitimacy and access to resources; re. shared strategic goals - Harris 'master plan' was a positive influence on creating 'collaborative resilience' but lack of core organisational mandate allowed attention to wane when operational issues were pressing (cf. link to internal credibility).
