# Supplementary material for: Governance of Intersectoral Collaborations for Population Health and to Reduce Health Inequalities in High-Income Countries: A Complexity-Informed Systematic Review
Source: Int J Health Policy Manag. 2022 Feb 23;11(12):2780–92. doi: 10.34172/ijhpm.2022.6550 (PMC10105187; doi:10.34172/ijhpm.2022.6550)
Supplement: Supplementary file 3 — Included Empirical Evaluative Studies and Their Characteristics. [file ijhpm-11-2780-s003.pdf]

Article title: Governance of Intersectoral Collaborations for Population Health and to Reduce Health Inequalities in High-Income Countries: A Complexity-Informed Systematic Review

Journal name: International Journal of Health Policy and Management (IJHPM)

Authors' information: Elizabeth Such[1]*, Katherine Smith[2], Helen Buckley Woods[3], Petra Meier[4]

[1]School of Health and Related Research, University of Sheffield, Sheffield, UK.

[2]University of Strathclyde, Glasgow, UK.

[3]Research on Research Institute, University of Sheffield, Sheffield, UK.

[4]MRC/CSA Social and Public Health Sciences Unit, Institute of Health and Wellbeing, University of Glasgow, Glasgow, UK.

(*Corresponding author: Email: e.such@sheffield.ac.uk)

Supplementary file 3. Included Empirical Evaluative Studies and Their Characteristics

Authors	Year	Title	Setting	Study design/methods	Sample(s) & methodological detail
Baum et al.	2015	New norms new policies: Did the Adelaide Thinkers in Residence scheme encourage new thinking about promoting well-being and Health in All Policies?	South Australia	Mixed methods<br>1. Semi-structured interviews<br>2. Evaluation workshops<br>3. Documentary analysis	34 with government/agency officials; 36 semi-structured interviews<br>Workshop 1 – 9 participants<br>Workshop 2 – 16 participants<br>Documents n=16
Baum et al.	2019	To what extent can the activities of the South Australian Health in All Policies initiative be linked to population health outcomes using a program theory-based evaluation?	South Australia	Mixed methods<br>1. Key informant interviews<br>2. Two electronic surveys<br>3. Documentary analysis	Interviews n=144<br>Survey of public servants: 2013 (n = 435) and 2015 (n = 483)<br>Analysis of state government policy documents
Carlisle	2010	Tackling health inequalities and social exclusion through partnership and community engagement? A reality check for policy and practice aspirations from a Social Inclusion Partnership in Scotland	Scotland, UK	Ethnographic design: observations and interviews	Detailed fieldnotes of management board meetings and other partnership events<br>Interviews with partnership members from statutory, voluntary and community sectors n=11

Cheadle et al.	2011	The impact of a community-based chronic disease prevention initiative: evaluation findings from Steps to Health King County.	Washington State, US	Mixed methods: Structured case study using a participatory approach<br>1. Analysis of routine data<br>2. Semi-structured key informant interviews<br>3. Documentary analysis	Pre-post evaluation of outcomes for 8 programmes. Other sample sizes/details not provided
Delany et al.	2016	Health in All Policies in South Australia: what has supported early implementation?	South Australia	Mixed methods<br>1. Interviews<br>2. Workshops<br>3. On-line survey	Interviews with public servants (n=45), politicians & political staff members (n=4), academics (n=3). Total of 62 interviews;<br>Workshop 1 – public servants (n=9)<br>Workshop 2 – public servants (n=16)<br>Survey – public servants n=168
Evenson et al.	2012	Evaluation of Physical Projects and Policies from the Active Living by Design Partnerships	US	Mixed methods<br>1. Key informant interviews<br>2. Focus groups<br>3. Web-based tracking system (routing data monitoring system)	Not detailed in paper. Available from Brennan LK, Brownson RC, Hovmand P. Evaluation of Active Living by Design: implementation patterns across communities. *Am J Prev Med* 2012;43(5S4):S351–S366.
Gase et al.	2014	Estimating the costs and benefits of providing free public transit passes to students in Los Angeles County: Lessons learned in applying a health lens to decision-making	Los Angeles County, US	Illustrative in-practice case example	Practitioner accounts and reflections on Health Impact Assessment process
Haigh et al.	2015	What makes health impact assessments successful? Factors contributing to effectiveness in Australia and New Zealand.	Australia and New Zealand	Mixed methods<br>1. Documentary analysis<br>2. Survey<br>3. Interviews<br>4. Depth case studies<br>5. Stakeholder workshops	Phase 1 Health Impact Assessment reports (n=55)<br>Phase 2 Survey (from 48 HIAs); follow-up interviews (n=34)<br>Phase 3 Case studies (n=11); key informant interviews (n=33); documentary analysis<br>Phase 4 Synthesis Research triangulation meeting; stakeholder validation workshop (n=77)
Harris et al.	2014	Housing, health and master planning: Rules of engagement	Western Sydney, Australia	Single explanatory case study<br>Documentary analysis<br>Interviews	Analysis of meeting documentation, reporting, written correspondence, practitioner reflections, stakeholder interviews (n=6) with stakeholders
Holt et al.	2018	Time to dismiss the idea of a structural fix within government? An analysis of	Danish municip-alities	Qualitative design<br>Interviews	Civil servant interviews (n=49) from 10 municipalities

		intersectoral action for health in Danish municipalities			
Holt et al.	2018	Elusive implementation: an ethnographic study of intersectoral policymaking for health.	Danish municip-alities	Ethnography	Single municipality.<br>Ethnographic field notes from participant observation (one year) and semi-structured (n=11) and informal interview transcripts
Hunter & Perkins	2012	Partnership Working in Public Health: The Implications for Governance of a Systems Approach	England, UK	Mixed methods<br>Interviews	Nine localities in England (2007-10)<br>Stage 1 – systematic literature review<br>Stage 2 – nine case studies; Semi structured interviews (phase 1 n=53 senior managers & elected officials; phase 2 n=8 Directors of Public Health; phase 3 n=32 frontline practitioners) & focus groups with service users (n=4)
Kelly et al.	2019	Increasing Opportunities for Health in a Southeast Michigan Community Through Local Policy Change.	Southeast Michigan, US	Single explanatory case study<br>Mixed methods – Interviews, analysis routine documentation/data	Interviews with partnership members (n=12)<br>Follow up interviews with 24 months between baseline and follow-up<br>Tracking forms, written agreement, job postings, reporting forms
Kokkinen et al.	2019	How and why do win–win strategies work in engaging policy-makers to implement Health in All Policies? A multiple-case study of six state- and national-level governments	Six countries	Explanatory multiple-case study<br>Key informant interviews<br>Literature review	n=10-15 interviews in each of the six case study sites<br>Peer-reviewed and grey literature
Lachance et al.	2018	Lessons Learned From Food & Fitness About Building Successful Partnerships: Focus, Capacity, and Sustainability.	US	Analysis of routine data	Six partnerships in diverse communities<br>Qualitative analysis of systems and policy change tracking form data over 9-year time period (2009-2016)
Lachance et al.	2018	The Food & Fitness Community Partnerships: Results From 9 Years of Local Systems and Policy Changes to Increase Equitable Opportunities for Health	US	Analysis of routine data	Six partnerships in diverse communities<br>Qualitative analysis of tracking form data over 9-year time period (2009-2016)
Langeveld et al.	2016	Use of a knowledge broker to establish healthy public policies in a city district: a developmental evaluation.	Amsterdam, The Netherlands	Action research<br>Participant observation	Knowledge broker as participant observer; data – field notes from observations including reflective journal
Lawless et al.	2012	Health in All Policies: evaluating the South Australian approach to intersectoral action for health.	South Australia	Qualitative design<br>Interviews	Semi-structured group and individual interviews policy staff, middle managers, executive-level staff and university researchers (n=31)

Mathias & Harris-Roxas	2009	Process and impact evaluation of the Greater Christchurch Urban Development Strategy Health Impact Assessment.	Greater Christchurch New Zealand	Qualitative design<br>1. Interviews<br>2. Focus groups<br>3. Questionnaires<br>4. Participant observation<br>5. Documentary analysis	Participant observation of HIA workshops (n=4) & survey of workshop participants<br>Focus group (n=1)<br>Documentary analysis of working group minutes, key reports & background documents<br>Key informant interviews (n=20)<br>Additional workshop (n=14)
McPherson et al.	2017	A catalyst for system change: a case study of child health network formation, evolution and sustainability in Canada.	Canada (rural settings)	Explanatory qualitative case study<br>Documentary analysis<br>Interviews	Case study of 13 year lifespan of Network<br>Review of documents (n=127)<br>Individual interviews (n=34)
Molnar et al.	2016	Using Win-Win Strategies to Implement Health in All Policies: A Cross-Case Analysis	Sweden, Quebec, South Australia	Explanatory multiple case study<br>Review of literature<br>Interviews	Telephone interviews with key informants (n=42)
Morteruel et al.	2020	Health Impact Assessments in Spain: Have They Been Effective?	Spain	Qualitative design<br>Interviews<br>Documentary review<br>Nonparticipant observation	Individual interviews (n=14)<br>Evaluation of 5 Health Impact Assessments
Plochg et al.	2013	Health governance by collaboration: a case study on an area-based programme to tackle health inequalities in the Dutch city of the Hague	The Hague, The Netherlands	Single prospective case study<br>Interviews<br>Documentary review	Semi-structured interviews with key informants (n=22)<br>Documents (n=7)
Politis et al.	2017	Pathways to policy: Lessons learned in multisectoral collaboration for physical activity and built environment policy development from the Coalitions Linking Action and Science for Prevention (CLASP) initiative	Canada	Qualitative design<br>Knowledge products analysis<br>Interviews	Review of knowledge products (n=183)<br>Key informant interviews (n=8)
Pursell & Kearns	2013	Impacts of an HIA on inter-agency and inter-sectoral partnerships and community participation: lessons from a local level HIA in the Republic of Ireland.	Ballyfermot, Ireland	Qualitative design<br>Interviews	Key informant interviews (n=21) from HIA steering group and community organisations involved in HIA. Mixture of face-to-face, telephone, group interviews and email
Sadare et al.	2020	Implementation of the Health Equity Impact Assessment (HEIA) tool<br>in a local public health setting: challenges, facilitators, and impacts	County of Simcoe and District of Muskoka, Canada	Survey questionnaire	Purposive sample<br>Administered online<br>n=40

Sestoft et al.	2014	The police, social services and psychiatry cooperation in Denmark A new model of working practice between governmental sectors. A description of the concept, process, practice and experience	Danish municipal-ities	Qualitative design<br>1. Interviews<br>2. Focus groups<br>3. Observations	Four selected municipalities in Denmark<br>Structured interviews (n=46)<br>Focus group discussions (n=2)
Steenbakkers et al.	2012	Challenging Health in All Policies, an action research study in Dutch municipalities.	Danish municipal-ities	Action research<br>Questionnaires<br>Logbook registry analysis<br>Interviews	Nine municipalities in Denmark<br>On-line questionnaires pre- & post-test with comparator with no intervention (n=119)<br>In-depth interviews (n=13) with public health municipal managers (8 action; 5 control)<br>Log book of activities by all participants in intervention
van Eyk et al.	2017	Health in All Policies in South Australia-Did It Promote and Enact an Equity Perspective?.	South Australia	Mixed methods<br>1. Interviews<br>2. Observation<br>3. Case studies<br>4. Documentary analysis<br>5. Workshops	Workshops (n=2)<br>Interviews (n=144) with Health Department policy actors, partner agencies, local government, political actors and researchers<br>Documents (n=5)<br>Case studies (n=5)
van Eyk et al.	2019	Creating a whole-of-government approach to promoting healthy weight: What can Health in All Policies contribute?	South Australia	Mixed methods<br>1. Semi-structured interviews<br>2. Document analysis<br>3. Program logic model	Documents (n=113)<br>Semi-structured interviews (n=31)
van Eyk et al.	2020	Improving child literacy using South Australia's<br>Health in All Policies approach	South Australia	Mixed methods<br>1. Document analysis<br>2. In-depth interviews<br>3. Program logic model	Documents (n=71)<br>In-depth interviews (n=7) with senior policy actors
